# *Porphyromonas gingivalis* and Human Cytomegalovirus Co-Infection: A Potential Link Between Periodontal Disease and Oral Cancer Development

**DOI:** 10.3390/cancers17091525

**Published:** 2025-04-30

**Authors:** Rancés Blanco, Juan P. Muñoz

**Affiliations:** 1 Independent Researcher, Av. Vicuña Mackenna Poniente 6315, La Florida 8240000, Chile; 2 Laboratorio de Bioquímica, Departamento de Química, Facultad de Ciencias, Universidad de Tarapacá, Arica 1000007, Chile

**Keywords:** human cytomegalovirus, *Porphyromonas gingivalis*, co-infection, periodontal disease, oral cancer

## Abstract

Periodontal disease (PD) is an inflammatory condition that can contribute to the development of oral cancer. Chronic inflammation from PD can lead to the release of inflammatory mediators and growth factors that promote tumorigenesis. *Porphyromonas gingivalis* (*P. gingivalis*) is one of several pathogens implicated in PD and its potential link to oral cancer. However, other viral infections, such as human cytomegalovirus (HCMV), can also contribute to chronic inflammation, creating a favorable environment for oral cancer development. Objectives: The present literature review tries to investigate the possible influence of *P. gingivalis* and HCMV co-infection in fostering the development of oral cancer and chronic periodontitis. Methods: A comprehensive search was conducted in PubMed and Google Scholar, focusing on the relevance and significance of articles that examine the role of *P. gingivalis* and HCMV in periodontal disease and oral cancer. Results: The evidence suggests that *P. gingivalis* and HCMV may act synergistically to modulate host immunity, disrupt epithelial integrity, and interfere with key cellular pathways. These interactions may enhance tissue destruction and foster a microenvironment conducive to malignant transformation. However, most of these findings stem from in vitro models and small-scale clinical studies, limiting the generalizability and clinical relevance of current conclusions. Conclusions: Although the proposed interaction between *P. gingivalis* and HCMV provides a compelling framework for understanding how microbial co-infections may influence oral cancer, the evidence remains preliminary and largely associative. To support these mechanistic hypotheses, future studies should give top priority to in vivo models, bigger patient cohorts, and longitudinal clinical studies.

## Simple Summary

Periodontal disease (PD) is a long-term inflammatory condition associated with oral cancer. Persistent inflammation in PD can drive tumorigenesis by triggering the release of inflammatory mediators and growth factors. *Porphyromonas gingivalis* (*P. gingivalis*), a major periodontal pathogen, and human cytomegalovirus (HCMV) may contribute to oral cancer by promoting immune dysregulation and chronic inflammation. This review examines the potential impact of *P. gingivalis* and HCMV co-infection on PD and oral cancer. A comprehensive literature search using PubMed and Google Scholar identified relevant studies. The evidence suggests that these pathogens may work together to sustain inflammation, interfere with immune responses, and alter essential cellular processes such as proliferation, apoptosis, and epithelial-to-mesenchymal transitions, ultimately driving cancer progression.

## 1. Introduction

Oral cancer (OC) encompasses a variety of tumors that arise in the oral cavity, including the lips, the anterior two-thirds of the tongue (oral tongue), buccal mucosa, the floor of the mouth, the retromolar trigone, and the soft and hard palates [1,2]. In 2022, oral cancer was the 16th most frequently diagnosed malignancy worldwide, with an estimated 389,485 new cases and 188,230 cancer-related deaths attributed to this disease [3]. According to the GLOBOCAN estimation, OC will reach the number of 641,563 new cases and 315,886 will be attributable to this disease by 2050, which represent an increase of 64.6% and 67.6%, respectively, compared to 2022. In terms of incidence, although an increase is anticipated across all human development index (HDI) tiers, the expected rise by 2050 varies significantly. Low HDI countries are projected to achieve an increase of approximately 147.8%, while medium HDI countries have a 94.2% projected increase, high HDI countries have a 67.3% projected increase, and very high HDI countries have only a 34.3% projected increase [4]. The socio-demographic disparities in the burden of OC highlight the critical need for public health initiatives that enhance disease prevention and early detection, improve healthcare access, and encourage lifestyle changes, particularly among high-risk populations.

Oral squamous cell carcinoma (OSCC) is the most common type of oral cancer, representing more than 90% of all epithelial tumors [1,2,3,5]. Several risk factors have been associated with the development of oral cancer, including tobacco use, alcohol consumption, poor oral hygiene, diet, human papillomavirus (HPV) infection, and chronic inflammatory conditions [6,7]. Premalignant lesions such as leukoplakia, erythroplakia, lichen planus, actinic cheilitis, and submucous fibrosis are also considered significant risk factors for oral cancer development [8].

As a chronic inflammatory disorder, periodontal disease (PD) compromises the gums, periodontal ligament, cementum, and alveolar bone—all of which are structures meant to support the teeth [9]. The main cause of this disorder is dental plaque, a microbial biofilm developing on tooth surfaces [10]. Usually split into two main forms, PD is gingivitis—superficial gum inflammation—or periodontitis, deeper inflammation marked by the progressive destruction of tooth-supporting tissues [11]. While radiographic imaging often shows symptoms such bleeding gums, the development of periodontal pockets, the deterioration of alveolar bone, and in advanced cases, tooth loss, clinical attachment loss (CAL) is used to evaluate the degree of periodontitis [10,12]. Although younger people can occasionally show aggressive forms of periodontitis, the chronic type is more common among adults [13,14]. Emphasizing its relevance as a common public health concern, data from the Global Burden of Disease (GBD) study show that severe chronic periodontitis affected almost 1.07 billion people between 1990 and 2021 [15].

PD has been associated with an increased risk of oral cancer, particularly OSCC [16,17,18]. The chronic inflammation of the periodontium can lead to the release of reactive oxygen species (ROS) and reactive nitrogen species (RNS), which induce oxidative stress and DNA damage [19]. ROS and RNS modulate the activity of the signal transduction pathways and diverse transcription factors involved in cancer initiation and progression, including mitogen-activated protein kinases (MAPKs), phosphoinositide 3-kinase (PI3K)/AKT, protein kinase C (PKC), NF-κB, Nrf2, STAT, HIF-1α, APE1/Ref-1, FOXO, AP-1, p53, and β-catenin [20]. Additionally, it produces a microenvironment where pro-inflammatory cytokines, the tumor necrosis factor-alpha (TNF-α), interferon-gamma (INF-γ), and other growth factors that support angiogenesis and survival are released by inflammatory cells like macrophages and lymphocytes [21]. Furthermore, tissue damage may make the oral mucosa more susceptible to the effects of other carcinogens such as tobacco, alcohol, and additional infections with other pathogens [22]. In addition, inflammatory processes can significantly alter a host’s immune response, leading to immune evasion and potentially favoring tumor initiation.

*Porphyromonas gingivalis* (*P. gingivalis*) is a gram-negative, non-motile, asaccharolytic, and anaerobic bacterium that plays a significant role in the development and progression of PD [23]. *P. gingivalis* possesses several virulence factors that contribute to its pathogenicity in PD, such as lipopolysaccharides (LPSs), outer membrane vesicles (OMVs), hemolysin, hemagglutinins, fimbriae, capsule, and gingipains [24]. It has been demonstrated that *P. gingivalis* can invade gingival epithelial cells, fibroblasts, and endothelial cells [25,26,27]. Inside the host cells, *P. gingivalis* can manipulate cellular processes, modulate the host immune response, and establish a persistent infection, which contributes to the tissue destruction and bone loss associated with the progression of PD [23]. The chronic inflammation induced by *P. gingivalis* can promote a microenvironment conducive to oral cancer development by inducing cellular stress and DNA damage.

The presence of *P. gingivalis* in an oral microbiome can also alter the overall microbial balance, potentially leading to dysbiosis, which is linked to cancer development [28]. *P. gingivalis* is frequently found in dental biofilms, where it interacts with other microorganisms, such as *Prevotella intermedia*, *Tannerella forsythia*, *Aggregatibacter actinomycetemcomitans*, and *Treponema denticola*, which are also known to be associated with severe periodontal conditions [29]. In the periodontium, *P. gingivalis* often coexists with certain viruses that potentially contribute to the pathogenesis of PD. For instance, some viruses, including herpes simplex virus-1 (HSV-1), Epstein-Barr virus (EBV), and human cytomegalovirus (HCMV), have been detected in subgingival samples from PD patients [30,31]. Consequently, it is plausible that interactions between *P. gingivalis* and these viruses may exacerbate the risk of oral carcinogenesis.

Human cytomegalovirus (HCMV), classified under the β-herpesviridae subfamily of the Herpesviridae family, is a ubiquitous pathogen with a global infection prevalence that may be approaching 90% [32]. The clinical presentation of an HCMV infection varies significantly depending on an individual’s immune function. In healthy individuals, a primary HCMV infection typically proceeds without noticeable symptoms, although in some cases it may mimic infectious mononucleosis [33,34]. In contrast, individuals with weakened immune defenses—such as organ transplant recipients or those living with AIDS—are particularly vulnerable to serious and potentially life-threatening complications associated with HCMV [35,36,37]. HCMV exhibits a broad tropism, allowing it to infect multiple human cell types, including monocytes, fibroblasts, smooth muscle cells, epithelial cells, and endothelial cells [38]. This wide cellular range supports the virus’s capacity to interfere with immune regulation through complex evasion strategies, effectively dampening host immune responses [39,40,41]. In cases of persistent infection, HCMV may contribute to a state of chronic inflammation, a condition associated with increased cancer risk in certain tissues [42]. Although a definitive link between HCMV and oral cancer has not yet been established, emerging evidence suggests several potential mechanisms through which the virus may influence oral carcinogenesis, which remain the subject of ongoing research.

The present literature review highlights the potential collaboration between *P. gingivalis* virulence factors and HCMV-encoded proteins in inducing chronic oral inflammation, evading a host’s immune response, and disrupting cellular functions, which may lead to the activation of oncogenic pathways. Additionally, the proposed model of *P. gingivalis* and HCMV interaction emphasizes the synergistic effect of these pathogens in driving the progression of PD and the initiation of oral cancer.

To investigate the impact of *P. gingivalis* and HCMV co-infection on the development of periodontal disease, a literature review of publications retrieved from the PubMed and Google Scholar databases up to 2 April 2025 was conducted. The literature search strategy and keywords are provided in Supplementary S1.

## 2. *P. gingivalis* and HCMV Co-Infection in Periodontal Disease

Studies have shown that a co-infection with *P. gingivalis* and HCMV may increase the risk of periodontitis, as the combined inflammatory responses can result in more extensive bone loss and soft tissue damage compared to infections with either pathogen alone [43,44,45].

Rafiei et al. (2017) [46] reported a statistically significant difference in the presence of *P. gingivalis* between periodontal patients and healthy controls (OR = 9.24, 95% CI: 5.78–14.77; *p* < 0.001). This study included 3356 patients with periodontal diseases and 2600 healthy individuals. The prevalence of *P. gingivalis* significantly increased from the periodontally healthy subjects (27.0%) to the gingivitis (37.5%) and chronic periodontitis patients (66.7%) (*p* < 0.05) [47]. Additionally, the median copy number of *P. gingivalis* was significantly higher in the chronic periodontitis patients (2.0 × 10^5^) compared to the chronic gingivitis (1.9 × 10^3^) and healthy control groups (8.8 × 10^2^) (*p* = 0.001) [48]. Moreover, the prevalence of *P. gingivalis* was strongly associated with probing pocket depth in chronic periodontitis patients (*p* < 0.001) [49]. Specifically, a higher frequency of *P. gingivalis* was detected in chronic periodontitis patients with probing pocket depth > 5 mm (93.3%) or ≤5 mm (60.0%) compared to the healthy subjects (10.0%) (*p* = 0.00002) [50]. Yong et al. (2015) [48] also found a relationship between the presence of *P. gingivalis* in gingival crevicular fluid and probing pocket depth (*p* < 0.001), bleeding index (*p* < 0.001), and clinical attachment loss (*p* = 0.007). 

In a meta-analysis by Botero et al. (2020) [51], which included 25 studies with 2385 participants, the risk of periodontitis was found to increase when a subgingival HCMV infection was detected (OR = 5.31, 95% CI: 3.15–8.97). Another meta-analysis based on case-control studies showed that the presence of HCMV was also significantly associated with aggressive periodontitis (OR = 3.63, 95% CI: 2.15–6.13; *p* = 0.009) [52]. Moreover, Zhu et al. (2015) [53] reported a significant association between HCMV infection and chronic periodontitis (OR = 3.59, 95% CI: 1.41–9.16; *p* = 0.007). Kazi and Bharadwaj [54] (2017) reported a higher prevalence of HCMV in patients with chronic periodontitis compared to healthy controls (19.3% vs. 0.3%, *p* < 0.001). HCMV infection was associated with the gingival index (*p* < 0.05), probing pocket depth (*p* < 0.001), and clinical attachment loss (*p* < 0.001) in chronic periodontitis patients. Joshi et al. (2015) [55] found HCMV DNA in 31% of chronic periodontitis tissues compared to 15% in healthy tissues (*p* = 0.011), which was also related to a higher plaque index (*p* = 0.015). Additionally, subgingival HCMV DNA was more prevalent in periodontitis patients compared to healthy individuals (43.3% vs. 14.3%, *p* = 0.028), with statistically significant differences observed in probing pocket depth and clinical attachment loss (*p* < 0.001) for both parameters [56].

The capacity of *P. gingivalis* to destroy crevicular tissues may facilitate a secondary HCMV infection of the epithelial cells in the periodontal pocket [43,44]. It was reported that HCMV-positive oral samples had significantly higher levels of *P. gingivalis* (*p* = 0.023) [55]. Furthermore, periodontitis was 7.6 times more likely to occur in patients with HCMV and a periodontopathic bacteria co-infection (OR = 7.6, 95% CI: 6.31–9.28; *p* < 0.01). In particular, an increased frequency of *P. gingivalis* was also detected in HCMV-positive aggressive periodontitis patients [57]. The co-presence of HCMV and *P. gingivalis* was significantly associated with localized juvenile periodontitis (OR = 51.4, 95% CI: 5.7–486.5), which was markedly higher compared to HCMV (OR = 4.6, 95% CI: 0.9–22.7) or *P. gingivalis* (OR = 7.8, 95% CI: 1.5–40.9) alone [43]. Furthermore, the co-presence of HCMV and *P. gingivalis* DNA was linked to periodontal pockets ≥ 4 mm with bleeding on probing (BOP) (*p* = 0.03) [44]. The odds of detecting HCMV in oral samples increased by 3.6 times when *P. gingivalis* was present (OR = 3.6, 95% CI: 1.5–8.8; *p* = 0.0054). Additionally, the number of teeth with attachment loss (≥2 mm) was significantly increased in juvenile periodontitis patients with the co-presence of HCMV and *P. gingivalis* in subgingival plaque [43]. The combined presence of *P. gingivalis* and HCMV increased the risk of aggressive periodontitis by 29.3-fold (OR = 29.3, 95% CI: 3.1–278.8; *p* = 0.003) [45]. Table 1 summarizes the studies related to the role of *P. gingivalis* and HCMV co-infection in periodontitis patients.

The co-presence of *P. gingivalis* and HCMV was evidenced in 12/30 (40.0%), 13/30 (43.3%), 6/30 (20.0%), and 7/30 (23.3%) of patients with generalized periodontitis, chronic periodontitis, gingivitis, and healthy individuals, respectively, without statistically significant difference among groups [31]. In addition, the co-presence of *P. gingivalis* and HCMV was associated with active periodontitis (*p* = 0.001) [61]. Whereas, Saygun et al. (2004) [59] also found an association of *P. gingivalis* and HCMV in aggressive periodontitis cases (*p* = 0.004). Moreover, Herrero-Sánchez et al. (2025) [60] reported the occurrence of *P. gingivalis* in 22/24 (91.7%) of the patients with periodontitis and only in 3/24 (12.5%) periodontally healthy individuals, which was statistically significant (*p* < 0.001). But, no HCMV infection was evidenced in these groups. Similar results were reported by Kousar (2017) [63]. Also, Sharma et al. (2015) [62] reported no synergistic correlation between the presence of *P. gingivalis* and HCMV in aggressive periodontitis patients (*p* = 0.732), while Passariello et al. (2017) [64] found an inverse association of *P. gingivalis* and HCMV occurrence in generalized aggressive periodontitis.

On the other hand, HCMV positivity was associated with the prevalence of *P. gingivalis* and *P. nigrescens* (OR = 3.23, 95% CI: 1.30–8.00; *p* = 0.01), *P. gingivalis*, *P. nigrescens*, and *T. denticola* (OR = 2.59, 95% CI: 1.00–6.68; *p* = 0.05), and *P. gingivalis*, *B. forsythus*, and *P. nigrescens* (OR = 3.23, 95% CI: 1.30–8.00; *p* = 0.01) [67]. In addition, Rams et al. (2024) [66] reported the occurrence of HCMV along with EBV-1 and subgingival *P. gingivalis*, *A. actinomycetemcomitans*, and other periodontopathic bacteria in 1/8 (12.5%) of aggressive periodontitis patients. Periodontitis sites displaying any combination of herpesviruses (HCMV, EBV-1 and HSV) showed a significantly higher occurrence of a *P. gingivalis* and *D. pneumosintes* co-infection compared to the sites showing no herpesvirus infection (*p* = 0.03) [65].

These findings together suggest a potential synergistic effect between *P. gingivalis* and HCMV in the development of periodontitis. However, despite some clinical studies indicating a frequent co-infection of *P. gingivalis* and HCMV in periodontal lesions [43,57,59], other studies report no association [60,62], highlighting inconsistent epidemiological findings and the need for larger, standardized clinical studies.

## 3. Association of Periodontal Disease and Oral Cancer

Research suggests a potential link between PD and an increased risk of OC development. Chronic inflammation and the presence of certain bacteria associated with PD may contribute to the carcinogenic process.

A meta-analysis conducted by Ma et al. (2024) [16], which included 6927 participants from five case-control studies, revealed a 2.94-fold increase in the risk of OC occurrence in patients with PD (OR = 2.94; 95% CI: 2.13–4.07; *p* < 0.001). Similarly, another meta-analysis published by Li et al. (2023) [18] found an association between PD and OSCC (OR = 3.28, 95% CI: 1.87–5.74). In addition, a positive association between severe PD and OSCC was reported (OR = 4.23; 95% CI: 2.92–6.13), but not when mild PD and OSCC were evaluated. The meta-analysis conducted by Ye et al. (2016) [17] also found a significant correlation between PD and OC risk (OR = 3.21, 95% CI: 2.25–4.16; *p* < 0.05). However, in general, these studies should be carefully interpreted due to heterogeneity or publication bias. Additionally, it was reported that the number of missing teeth related to periodontal problems was significantly higher in OSCC patients compared to the control group (40.0% vs. 25.9%; *p* = 0.001) [68].

Moreover, the risk of OSCC occurrence was increased in patients with severe periodontitis (aOR = 4.07, 95% CI: 1.50–11.03) and incipient periodontitis (aOR = 3.46, 95% CI: 1.35–8.90) compared with patients having a normal periodontal status, which was more evident among the patients with increased tooth loss (aOR = 9.99) [69]. The frequency of poorly differentiated OSCC was also higher in patients with periodontitis compared to those without periodontitis (32.8% vs. 11.5%; *p* = 0.038) [70]. Interestingly, Wen et al. (2014) [71] showed that patients with periodontitis exhibited a higher risk of OC development compared to those with gingivitis (aHR = 1.79, 95% CI: 1.42–2.25; *p* < 0.001).

The bone loss measures were associated with an increased risk of OC development (OR = 4.52; 95% CI: 3.03–6.75) [70] and (OR = 2.4; 95% CI: 3.04–11.7) [72]. The association of bone loss with OSCC occurrence was also radiographically demonstrated (OR = 2.4, 95% CI: 1.5–3.8; *p* < 0.001) [72]. Furthermore, alveolar bone loss (ABL) (per millimeter) was associated with an increased risk of OC (aOR = 4.52; 95% CI: 3.03–6.75) [70]. In particular, Tezal et al. (2009) [70] found an association between ABL and a 5.23-fold increased risk of tongue cancer development (OR = 5.23, 95% CI: 2.64–10.35). It was reported that patients with fewer than 5 teeth had a 2.4-fold increased risk of OC occurrence compared with patients with 15 or more teeth (OR = 2.4, 95% CI: 1.3–4.1) [73]. A significant link between the risk of OC and patients with >15 missing teeth was also reported by other authors (OR = 1.91, 95% CI: 1.01–3.62; *p* = 0.047) [16]. In addition, a positive association was observed between OC and the loss of more than 20 teeth (OR = 2.40, 95% CI: 0.97–5.97; *p* = 0.05) [74]. Remarkably, Zheng et al. (1990) [75] found that the loss of teeth without replacement is associated with an increased risk of OC development (OR = 3.7, 95% CI: 2.2–6.4, for males) and (OR = 8.3, 95% CI: 3.5–19.6, for females).

Komlós et al. (2021) [76] reported an increased incidence of OC in patients with PD compared with those without periodontitis (57.1% vs. 28.6%), which was also related to the periodontal stage. Moreover, there was an increased CAL, probing pocket depth (PPD), Silness–Löe plaque index, and decayed, missing, and filled teeth (DMFT) index in patients with OC compared to the control group [76]. Furthermore, a significant increase in CAL (SMD = 1.94; 95% CI: 0.22–3.66; *p* = 0.027) and DMFT (SMD = 0.65, 95% CI: 0.12–1.18; *p* = 0.017) was observed in the OC patient group compared to the control group [16]. All these facts together suggest an association between PD (as indicated by periodontal status) and OC development.

## 4. Frequency of *P. gingivalis* and HCMV Infections in OSCC

Some studies have found an increased frequency of *P. gingivalis* and HCMV infections in OSCC, suggesting that both pathogens, separately, may play a role in the development and progression of these tumors [77,78,79].

An increased presence of *P. gingivalis* was found in OSCC samples compared to non-tumor tissues [77,78,80], although opposite results were also reported [81]. For instance, *P. gingivalis* was detected in 14/30 (46.6%) OSCC patients [80]. Additionally, *P. gingivalis* was evidenced in 10/10 (100%) of gingival SCC patients by immunohistochemistry (IHQ), while the immunostaining in the normal gingival control group was 33% less intense (*p* < 0.05) [82]. Moreover, an increased presence of *P. gingivalis* was reported in OSCC tissues compared to normal samples. *P. gingivalis* FimA genotypes found in saliva were consistent with those obtained from the respective OSCC tissue, suggesting that the salivary microbial reservoir could be the source of *P. gingivalis* in OSCC tissues [83].

It was also reported that *P. gingivalis* detection by fluorescence in situ hybridization (FISH) significantly increased from normal tissues (4/30, 13.3%) to paracancerous samples (20/61, 32.8%) and OSCC (37/61, 60.7%) (*p* < 0.05). The positive rate of *P. gingivalis* was higher in the OSCC patients with advanced clinical stages (III–IV), a decreased degree of tissue differentiation, and the occurrence of lymph node metastasis (*p* < 0.05) [77]. Similarly, *P. gingivalis* was detected in 119/205 (58.0%) OSCC samples by IHQ. This immunoexpression was linked to poor differentiation, increased tumor size, advanced clinical stage, and decreased overall survival of OSCC patients. Additionally, the presence of *P. gingivalis* in OSCC tissues was associated with the expression of CXC motif chemokine ligand 2 (CXCL2) and tumor-associated neutrophils (TANs) [78]. *P. gingivalis* infection was detected in 22 of 56 (39.3%) OSCC samples by IHC and was linked to a reduced overall survival of patients [84].

On the other hand, the prevalence of HCMV infection in OSCC varies globally, ranging from 5.17% to 84.0% [85,86,87], although some studies have reported a lack of HCMV infection in these tumors [88,89,90]. Specifically, Naqvi et al. (2020) [85] found HCMV DNA in 3/58 (5.17%) OSCC samples. Similarly, Saravani et al. (2015) [91] demonstrated HCMV DNA positivity in 6.3% of OSCC samples, with an average viral load of 57.7 × 10^3^. HCMV DNA was detected in 6/26 (23.07%) of head and neck squamous cell carcinoma (HNSCC) cases, including those from the buccal mucosa and lips [92]. In addition, HCMV DNA was found in 22/60 (36.6%) OSCC cases. Interestingly, the presence of HCMV correlated with higher grades and histological stages of OSCC [93]. A positive in situ hybridization (ISH) reaction for HCMV DNA was reported in 26/60 (43.3%) OSCC samples, which was statistically significantly higher compared to the control group [87].

An immunohistochemical study conducted by Khashman (2017) [86] demonstrated the expression of HCMV pp65 protein in both OSCC and healthy individual groups. The expression of this viral protein was found in 21/25 (84.0%) OSCC samples, which was significantly higher compared to the tissue samples from healthy individuals (7/17, 41.2%) (*p* = 0.0038) [86]. HCMV DNA was also detected in 21% of the OSCC samples and 2% of the non-tumor controls. In this study, it was further shown that HCMV infection increased the probability of OSCC development by 13.02 times (OR 13.02, 95% CI: 2.96–57.2; *p* = 0.007). Moreover, HCMV infection was associated with more advanced stages of the disease (*p* = 0.003) [79]. In another study HCMV detection by ISH was found in 17/40 (42.5%) OSCC samples, which was significantly higher compared to benign oral tumors (10/40, 25%) and healthy tissues (3/40, 7.5%) (*p* < 0.001). The development of OSCC was shown to be 12.7 times more likely in the presence of HCMV (OR 12.7, 95% CI: 2.6–54.1) [94].

These studies suggest that co-infection with *P. gingivalis* and HCMV could occur in OSCC and may contribute to the development and progression of these tumors.

## 5. Mechanisms of *P. gingivalis* and HCMV That Induce Immune Evasion and Chronic Inflammation

### 5.1. Disruption of Host Immune Response

The disruption of Toll-like receptor (TLR) recognition and the subsequent production of pro-inflammatory cytokines are critical initial steps in immune evasion. In this regard, *P. gingivalis* evades TLR4 sensing by modifying the lipid A region of its LPS to an immunologically silent lipid A [95]. The *P. gingivalis* LPS induces the expression of interleukin (IL)-1R-associated kinase (IRAK)-M in macrophages, which acts as a negative regulator of the TLR signaling pathway [96]. Whereas, HCMV encodes US7 and US8 proteins, which degrade TLR3 and TLR4 [39]. Additionally, HCMV microRNA (miR)-UL112-3p inhibits TLR2 and the production of IL-1β, IL-6, and IL-8 cytokines, modulating the TLR2/IRAK1/NFκB signaling pathway [40]. Also, *P. gingivalis* gingipains degrade a variety of immune mediators such as the coreceptor of TLRs, CD14 [97], antimicrobial peptides (e.g., LL-37) [98], the molecules of the complement system (e.g., C3, C4, and C5) [99], cytokines (e.g., IL-1β, IL-6, IL-8, IFN-γ, and TNF-α) [100,101,102], cytokine receptors (e.g., IL-6R) [103], and immunoglobulins (e.g., IgG1 and IgG3) [104].

On the other hand, the penta-acylated lipid A structures of *P. gingivalis* LPSs activate the TLR4-mediated NF-*κ*B signaling pathway, inducing the production of pro-inflammatory IL-6 and IL-8 cytokines by human gingival fibroblasts [105]. In addition, *P. gingivalis* suppresses TLR2-induced IL-12p70 while simultaneously leveraging C5aR-TLR2 crosstalk to enhance the production of pro-inflammatory and bone-resorptive cytokines, including IL-1β, IL-6, and TNF-α [106]. Furthermore, *P. gingivalis* activates NLRP3 and AIM2 inflammasomes through caspase 1 recruitment, inducing the maturation and release of IL-1β [107]. While an HCMV infection increases the release of mitochondrial DNA (mtDNA) into the cytoplasm of monocyte cells, triggering the activation of the NLRP3 inflammasome and IL-1β maturation [108]. This dual role in modulating the production of pro-inflammatory cytokines may enable *P. gingivalis* and HCMV to sustain chronic inflammation, such as periodontitis, while evading effective immune elimination.

It has been reported that HCMV IE proteins induce the expression of ICAM-1 mRNA levels in human umbilical vein endothelial cells (HUVECs) [109]. Furthermore, HCMV UL23 inhibits the transcription of IFN-γ-stimulated genes and blocks antiviral IFN-γ responses by interacting with the human N-myc interactor (Nmi) protein [41]. Whereas, the OMVs of *P. gingivalis* also induce the expression of ICAM-1 and E-selectin by vascular endothelial cells and inhibit IFN-γ-mediated MHC-II expression by these cells [110]. Remarkably, significantly decreased levels of IFN-γ and increased mortality rates were observed in murine CMV (MCMV) and *P. gingivalis* co-infected mice compared to those infected with MCMV or *P. gingivalis* alone [111]. This suggests potential cooperation between MCMV and *P. gingivalis* in disrupting the mice’s immune response, which may lead to increased infection persistence. Interestingly, *P. gingivalis* gingipains can also cleave ICAM-1 on oral epithelial cells, potentially disrupting the interaction of these cells with polymorphonuclear leukocytes [112].

*P. gingivalis* induces the expression of PD-L1 on dendritic cells (DCs) through the activation of Akt and STAT3 pathways, which in turn suppresses the activity of CD8+ T-cells [113]. Similarly, HCMV UL23 induces the upregulation of PD-L1 in human fibroblasts and also the evasion of T cell-mediated cytotoxicity [114]. Additionally, HCMV US2, US3, and US11 are involved in the downregulation of MHC-I heavy chains, which may disrupt immune recognition and promote viral persistence [115]. Moreover, HCMV US2 induces the degradation of two components of the MHC-II pathway (HLA-DR-α and DM-α), preventing the recognition of HCMV antigens by CD4+ T cells [116]. HCMV also decreases MHC-II regulation of the expression of the class II transactivator (CIITA) [117]. Since both MHC-I and MHC-II are essential for bacterial antigen presentation, the inhibition of their expression by HCMV may facilitate the escape of *P. gingivalis* from immune surveillance.

### 5.2. ROS Production and Other By-Products Related to Tissue Damage 

ROS play a significant role in the pathophysiology of periodontitis. During an inflammatory response, immune cells such as neutrophils and macrophages generate ROS as a defense mechanism against pathogens. Additionally, certain periodontopathic bacteria, including *P. gingivalis*, can also contribute to ROS production through their metabolic processes [19]. In fact, *P. gingivalis* can induce ROS production and JAK2 activation in gingival epithelial cells, which in turn upregulates the production of the pro-inflammatory cytokines IL-1β and IL-6 through c-Jun activation [118]. Furthermore, the LPSs from *P. gingivalis* can stimulate human gingival fibroblasts to increase mitochondrial ROS (mtROS) production, leading to the secretion of pro-inflammatory cytokines [119]. An increased level of ROS and the expression of the HK2 glycolysis-related gene were also observed in human gingival fibroblasts exposed to *P. gingivalis*, which was associated with the induction of an inflammatory response [120]. During the early phase of infection, HCMV is capable of inducing ROS production in human smooth muscle cells [121,122]. HCMV-induced ROS was partially linked to the activation of the cyclooxygenase-2 (COX-2) pathway [121]. Additionally, H_2_O_2_, the most common type of ROS, induces the HCMV replication in human foreskin fibroblast cells through the activation of the p38-MAPK signaling [123]. These findings suggest that ROS production induced by *P. gingivalis* could favor HCMV replication in oral tissues. Excessive ROS production also leads to the formation of free radicals that can damage cellular components such as nucleic acids, lipids, and proteins, resulting in tissue damage and exacerbating chronic inflammation [124].

On the other hand, *P. gingivalis* produces several metabolites that influence immune responses, promote chronic inflammation, and tissue damage, including short-chain fatty acids (SCFAs), hydrogen sulfide (H2S), amino acid metabolites, and indole derivatives [125,126]. For example, *P. gingivalis* can form methanethiol (methyl mercaptan) from L-methionine [127], which is cytotoxic to human gingival fibroblasts [128]. In addition, *P. gingivalis* can produce indole from tryptophan, which is associated with promoting dysbiotic periodontal biofilm formation, bone resorption, and tissue inflammation [129]. The production of volatile sulfur compounds, such as hydrogen sulfide, methanethiol, and dimethyl disulfide by *P. gingivalis*, increases the permeability of oral mucosa and enhances bacteria invasiveness [22]. Meanwhile, an HCMV infection stimulates lipid peroxidation [130], which is elevated in periodontitis patients compared to healthy individuals [131]. Moreover, an HCMV infection induces peroxynitrite production (an RNS), which, in turn, promotes viral replication in human fibroblasts [132]. Notably, it has been demonstrated that peroxynitrite activates pro matrix metalloproteinase (proMMP)-8 [133], suggesting its potential role in chronic inflammation and periodontium destruction.

In addition to sustaining chronic inflammation, oxidative stress plays a direct role in oral carcinogenesis by inducing genetic mutations and promoting genomic instability. ROS, such as hydroxyl radicals and hydrogen peroxide, can damage DNA bases, leading to mutations, particularly the G to T transversions commonly observed in the *TP53* tumor suppressor gene in OSCC [134]. The oxidative DNA lesion 8-hydroxydeoxyguanosine (8-OHdG) has been frequently detected at elevated levels in oral cancer tissues and is considered a reliable biomarker of oxidative damage [135]. Moreover, ROS affect various stages of carcinogenesis: they initiate tumor formation by causing DNA mutations, promote tumor growth through the inhibition of apoptosis, and support cancer progression via the activation of transcription factors such as NF-κB and HIF-1α [134]. These factors not only enhance survival signaling but also contribute to angiogenesis and metastasis. Therefore, chronic microbial infections, like those induced by *P. gingivalis* and HCMV, may contribute to this oxidative environment by activating host immune responses and producing ROS directly, thereby synergizing these mutagenic processes in oral cells.

### 5.3. Disruption of the Blood Coagulation System

The disruption of the blood coagulation system, particularly through excessive fibrin deposition, platelet activation, and the upregulation of coagulation factors, plays a critical role in the persistence of chronic inflammation, which in turn can lead to further tissue damage and potentially oral cancer [136].

The gingipains produced by *P. gingivalis* have been shown to possess the ability to cleave and activate coagulation factors IX, X, and prothrombin. [137,138,139]. Neilands and Kinnby (2022) [140] also demonstrated an association between the procoagulant activity of *P. gingivalis* gingipains and fibrin formation [140]. Extravascular fibrin deposition induces the expression of pro-inflammatory factors by endothelial cells and peripheral blood mononuclear cells, as well as the activation of inflammation-related pathways (e.g., NF-κB, MAPK, and PKC), which may contribute to the development of periodontitis [141,142]. The infection of endothelial cells with HCMV induced the expression of the von Willebrand factor, which is associated with platelet adhesion and aggregation [143]. Moreover, HCMV is also able to upregulate the expression of the adhesion molecules LFA-3 and ICAM-1 on the surface of infected fibroblasts, increasing the adhesion of peripheral blood leucocytes to these cells [144,145]. Notably, it has been suggested that platelet activation can stimulate matrix metalloproteinase (MMP)-1 production by gingival fibroblasts, promoting periodontal tissue degradation [146].

Furthermore, it has been demonstrated that *P. gingivalis* LPSs induce the expression of IL-17 in THP-1 cells, which is linked to an increased production of thrombospondin-1 (TSP-1) [147]. The increased production of TSP-1 may protect *P. gingivalis* from neutrophil bactericidal activity, contributing to the progression of PD [148]. Additionally, TSP-1 can promote *P. gingivalis* LPS-induced extracellular matrix degradation and alveolar bone destruction through the activation of the p38 MAPK signaling pathway [149]. On the other hand, HCMV induces the production and secretion of the plasminogen activator inhibitor type I (PAI-1) by endothelial cells [150]. PAI-1 regulates the conversion of plasminogen to plasmin and plays a significant role in inflammatory processes [151]. *P. gingivalis* is also capable of inducing PAI-1 production in human aortic endothelial cells [152]. However, the lysine-specific gingipain-K (Kgp) from *P. gingivalis* can degrade PAI-1, which may delay wound healing in endothelial cells [153].

**Figure 1:**
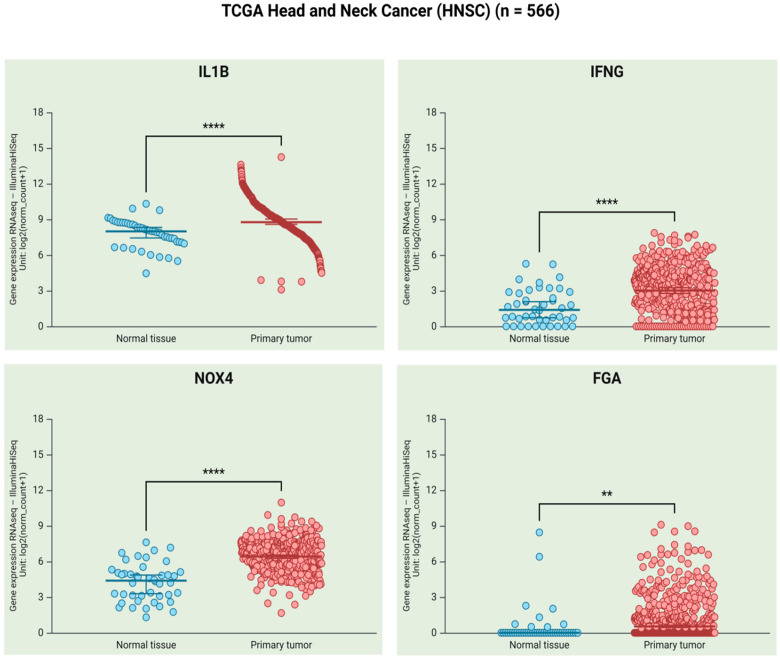
Levels of IL1B, IFNG, NOX4, and FGA transcripts in primary head and neck cancer and normal tissues (TCGA HNSC, *n* = 566 after removing samples with nulls). The levels of all mRNAs were significantly increased in primary tumors compared to normal samples (**** *p*  < 0.0001, ** *p*  <  0.01; statistical significance was determined using Welch’s *t*-test). The raw data were obtained from the University of California, Santa Cruz (UCSC) genome database https://xena.ucsc.edu (accessed on 6 March 2025). For the correlation of IL1B, IFNG, NOX4, and FGA expression levels and type of samples, the UCSC Xena platform was used (https://xenabrowser.net, accessed on 6 March 2025). The graphs were created using Biorender.com.

Overall, the evidence suggests that co-infection with *P. gingivalis* and HCMV may play a role in the initiation and progression of PD by facilitating immune evasion, causing tissue damage, and promoting chronic inflammation. Moreover, the chronic inflammation of periodontium could promote oral carcinogenesis. In fact, according to the Cancer Genome Atlas (TCGA) database [154,155], the expression levels of some molecules related to immune evasion (IL1B and IFNG), a marker of oxidative stress (NOX4), and a protein involved in the coagulation cascade (FGA) are overexpressed in head and neck cancer, which includes OC (Figure 1).

### 5.4. Interactions of P. gingivalis and Other Viruses

*P. gingivalis* may also cooperate with other viruses, such as EBV, HPV, and HSV-1, to promote immune evasion and chronic inflammation. For instance, Shigeishi et al. (2022) [156] found an association, although not statistically significant, between the co-presence of *P. gingivalis* and EBV and a periodontal inflamed surface area, which is an indicator of the severity of periodontal inflammation (*p* = 0.08). *P. gingivalis*-produced butyric acid accumulation induces the transition of EBV from latency to the lytic replication cycle, increasing the transcriptional activity of the immediate-early BZLF1 gene [157]. Interestingly, the expression of the EBV BZLF-1 gene positively correlated with IL-1β or IL-6 mRNA expression in periapical granulomas [158]. Moreover, BZLF1 downregulates the expression of CD74, facilitating EBV evasion from CD4+ T cell responses during the lytic cycle [159].

Moreover, the treatment of HPV16-transfected oral keratinocytes with spent media from *P. gingivalis* induced an increased expression of the HPV16 E7 protein compared to cells treated with spent media from the commensal bacterium *Streptococcus sanguinis* [160]. Notably, it has been reported that HPV16 E7 can drive the immune escape of oral cancer cells through the nucleotide-binding oligomerization domain, leucine-rich repeat containing X1 (NLRX1)-mediated degradation of STING [161]. In addition, *P. gingivalis* end-products increase HPV16 E6/E7 expression in a p38-dependent manner and also induce ROS production in the HPV16-positive SiHa cells [160]. The capacity of HR-HPV E6/E7 proteins to dysregulate diverse cellular pathways that allow the virus to evade host immune defense is well known [162,163].

Finally, both an HSV-1 and an HSV-2 infection was significantly associated with severe periodontitis [164]. The in silico analysis suggested a potential interaction between the *P. gingivalis* KGP gingipain and HSV ICP4 protein, which is critical for viral replication [165]. It was demonstrated that HSV-1 infects human gingival fibroblasts and upregulates a variety of pro-inflammatory factors including interferon regulatory factor-3 (IRF3) and IRF7, myeloid differentiation factor 88 (MyD88), IFN-β1, TNF, chemokine (CXC motif) ligand 11 (CXCL11), IL-1α, IL-1β, gamma-IFN-inducible protein 16 (IFI16), CC motif ligand 5 (CCL5), and also TLR2, TLR3, and TLR7 [166]. The production of pro-inflammatory factors and cytokines that favor bone resorption may enable HSV to sustain chronic periodontitis, while cooperating with *P. gingivalis* persistence.

## 6. Oncogenic Properties of *P. gingivalis* and HCMV Proteins

The infection of oral cells with *P. gingivalis* and HCMV promotes proliferation, inhibits apoptosis, induces epithelial–mesenchymal transition (EMT) and cell migration, tumor formation, and the evasion of the host’s immune response. The molecular mechanisms by which *P. gingivalis* and HCMV trigger the initiation and progression of oral cancer are described below.

### 6.1. Activation of Signaling Pathways That Control Cell Proliferation

*P. gingivalis* and HCMV proteins can activate a variety of signaling pathways that regulate cell proliferation. In fact, *P. gingivalis* isolated from patients with chronic periodontitis induced increased proliferation and reduced the apoptosis of gingival epithelial cells compared to the non-infected cells, which was associated with the expression of the O-antigen region of *P. gingivalis* LPSs [167]. Kuboniwa et al. (2008) [168] demonstrated that the infection of gingival epithelial cells with *P. gingivalis* accelerates progression through the S-phase of the cell cycle, which involved the presence of *P. gingivalis* long fimbriae. Furthermore, it was reported that a *P. gingivalis* infection of immortalized human gingival epithelial (IHGE) cells upregulates the expression of cyclin D1 and cyclin E, increasing the proliferation rate by promoting the G1/S transition [169]. Whereas, HCMV UL97 phosphorylates Rb, p107, and p130, inactivating their ability to repress the E2F-responsive E2F1 promoter in Saos-2 cells [170,171]. Similarly, the HCMV IE1 interacts with p107, disabling its transcriptional repression on the E2F-responsive promoter and inducing the activity of cyclin E/CDK2 in C33A cervical cancer cells [172,173]. Moreover, an infection of breast epithelial cells with HCMV induces the activation of STAT3, which in turn leads to the upregulation of cyclin D1, increasing cell proliferation [174]. Additionally, the overexpression of cyclin D1 and its upstream transcription factor AP-1 (comprising c-Jun and c-Fos) were observed in Tca8113 oral cancer cells infected with *P. gingivalis*, contributing to increased proliferation rates compared with the control cells [175]. In another study, the treatment of breast epithelial cells with cmvIL-10, an IL-10 homologue encoded by HCMV, induced DNA synthesis and activated the Janus kinase 1 (JAK1)/STAT3 pathway, further increasing cell proliferation [176]. Furthermore, Huxiao et al. (2024) [177] reported increased proliferation and migration rates of oral carcinoma cells treated with *P. gingivalis* gingipain extract. Overall, *P. gingivalis* and HCMV proteins may synergize to disrupt the control of a cell cycle, promoting uncontrolled cell proliferation.

### 6.2. Regulation of Apoptosis and Cell Survival

*P. gingivalis* and HCMV may interfere with the regulation of programmed cell death and cellular stress responses. For instance, *P. gingivalis* induces the upregulation of the anti-apoptotic molecule Bcl-2 in GECs [178]. *P. gingivalis* also blocks mitochondrial depolarization and cytochrome *c* release in GECs, which are related to the activation of PI3K/Akt pathway [179]. In the same cells, it was also demonstrated that *P. gingivalis* activates the JAK/Stat pathway, which in turn induces the overexpression of surviving [180]. *P. gingivalis* activates the Akt signaling, which in turn induces the inactivation of the pro-apoptotic Bad. Furthermore, *P. gingivalis* can inhibit the pro-apoptotic caspase-9 (CASP9) independent of Akt activation [181]. The capacity of HCMV-encoded proteins such as IE1, IE2, and UL36-38, to prevent apoptosis in human epithelial cells was previously reviewed [182]. For example, HCMV UL36 binds to the pro-domain of caspase-8, preventing its activation and inhibiting Fas-mediated apoptosis [183]. Meanwhile, HCMV UL37 recruits Bax to mitochondria, blocking the apoptosis mediated by death receptors [184,185]. Moreover, some HCMV-encoded miRNAs, such as miR-UL36-3p, miR-US5-1, miR-US4-5p, miR-US25-2-3p, and miR-US4-3p, are capable of targeting and downregulating the expression of the pro-apoptotic FAS, FADD, caspase-2 (CASP2), caspase-3 (CASP3), and caspase-7 (CASP7), respectively [186]. It was also reported that hcmv-miR-UL70-3p targets the 3′UTR region of the modulator of apoptosis-1 (MOAP1) mRNA, downregulating the H2O2-induced apoptosis [187]. Together, these findings suggest that *P. gingivalis* and HCMV may collectively prevent apoptosis through diverse molecular mechanisms.

### 6.3. Induction of EMT and Invasiveness

EMT is considered a critical event in tumor invasion and metastasis [188]. Specifically, the switch from E-cadherin to N-cadherin indicates the EMT process and plays a significant role in both the invasion and metastasis of OSCC [189]. Huxiao et al. (2024) [177] reported decreased levels of E-cadherin in oral carcinoma cells exposed to *P. gingivalis* gingipain extract, while the expression levels of N-cadherin significantly increased. Moreover, chronic infection of OSCC cells with *P. gingivalis* decreased the expression of cytokeratin 13 (CK13) while increasing the expression of well-established EMT markers such as N-cadherin and α-SMA. In addition, P gingivalis was able to upregulate the expression of Snail, Slug, and Twist, which are associated with the suppression of epithelial cells features [190]. Similarly, the infection of colorectal cells with HCMV increased the expression of mesenchymal markers such as N-cadherin and fibronectin and also decreased E-cadherin. Furthermore, the expressions of other factors involved in the orchestration of EMT such as ZEB1, TWIST1, SNAIL1, and SNAIL2/SLUG were upregulated [191]. It was also found that the tumor samples from NOD/SCID gamma mice inoculated with HCMV-infected breast epithelial cells showed decreased levels of E-cadherin as well as cytokeratins CK5/6 and CK20, while vimentin expression increased [174]. The infection of human mammary epithelial cells with a high-risk HCMV strain also induced a reduction in the expression of the epithelial cell adhesion molecule (EpCAM) [192]. Furthermore, it was demonstrated that repetitive exposure to *P. gingivalis* increases the migration and invasiveness of OSCC cells [190]. Specifically, *P. gingivalis* gingipains were reported to cleave type I collagen [193]. Additionally, the role of *P. gingivalis* gingipains in the activation of proMMP9 was demonstrated [194]. Meanwhile, treatment with cmvIL-10 increased the MMP-3 mRNA levels and the invasiveness of breast cancer cells [176]. Taken together, the capacity of *P. gingivalis* and HCMV to induce EMT, proteolytically degrade of ECM components, and activate MMPs suggests the potential role of these pathogens in the acquisition of a mesenchymal phenotype by epithelial cells, thereby increasing their invasive properties.

### 6.4. Promotion of Cell Immortalization and Tumor Growth

**Figure 2:**
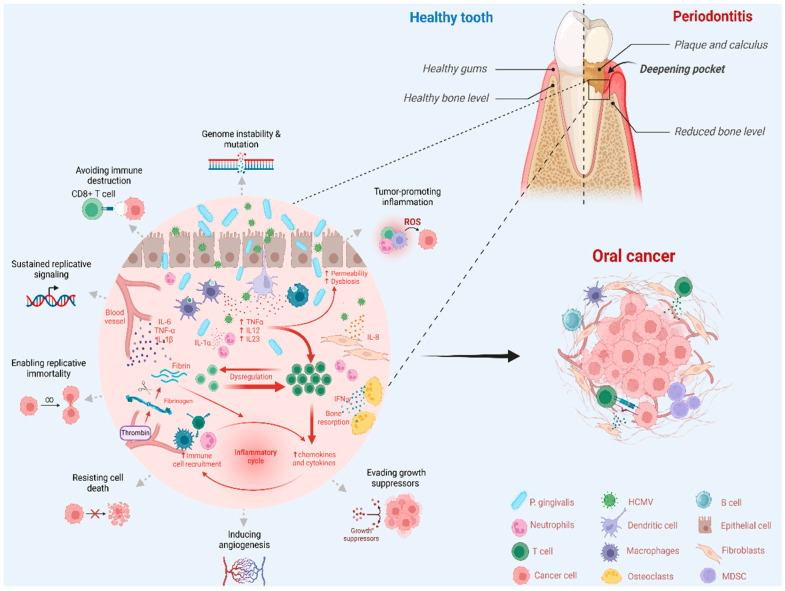
Hypothetical model of *P. gingivalis* and HCMV co-presence for periodontal disease and oral cancer development suggested in this proposal. Created by Biorender.com.

The activation of the catalytic subunit of human telomerase reverse transcriptase (hTERT) is crucial in tumor formation, maintaining telomere length, and enabling cells to evade senescence [195]. During the earlier phases of the replication cycle, HCMV can upregulate the expression of hTERT in human fibroblasts [196]. Moreover, increased hTERT expression and telomerase activity have been reported in human fibroblasts and glioblastoma cells infected with HCMV [196,197]. In addition, human astrocytes infected with a high-risk HCMV strain were able to form glioblastoma-like tumors in Ragγ2C^−/−^ mice [198]. Furthermore, the capacity of HCMV to induce the formation of non-differentiated tumors when infected human cells were transplanted into athymic nude mice was previously reported [199]. Kumar et al. (2018) [174] also found that human mammary epithelia cells infected with a high-risk HCMV strain could induce tumor formation in NOD/SCID gamma mice. While *P. gingivalis* was able to promote oral cancer progression in C57BL/6 mice, which was associated with its ability to recruit tumor-associated neutrophils (TANs) through the activation of the CXCL2/CXCR2 signaling in the tumor microenvironment [200]. In addition, *P. gingivalis* enhances the OSCC growth in NOD/SCID mice by inducing M2 tumor-associated macrophages (TAMs) polarization, which display protumor activities [201]. *P. gingivalis* invaded oral lesions and promoted tumor progression in a 4-nitroquinoline-1 oxide (4NQO)-induced carcinogenesis mouse model, a process also associated with the infiltration of oral lesions by myeloid-derived suppressor cells (MDSCs), which play an important role in tumor-mediated immunosuppression [84]. Taken together, the evidence suggests that the cooperation between *P. gingivalis* and HCMV may promote cell immortalization and oral tumor formation. A hypothetical model of *P. gingivalis* and HCMV co-presence for the development of OSCC is shown in Figure 2.

### 6.5. Potential Diagnostic Markers for Co-Infection-Related Oral Cancer

The early detection of OSCC in patients co-infected with *P. gingivalis* and human HCMV may benefit from the identification of specific molecular and microbial biomarkers. Elevated salivary levels of *P. gingivalis* DNA and HCMV DNA, detectable via qPCR, have been associated with periodontal inflammation and are proposed as non-invasive indicators of microbial burden [202,203]. Additionally, oxidative DNA damage markers such as 8-OHdG, which accumulate in the presence of the ROS generated by chronic infections, have been linked to early carcinogenic changes in oral epithelial cells [135]. Inflammatory cytokines such as IL-1β, IL-6, and TNF-α, along with immune checkpoint molecules like PD-L1, are often upregulated in the tumor microenvironment influenced by these pathogens and may serve as adjunct diagnostic or prognostic indicators. Moreover, an overexpression of EMT markers (e.g., N-cadherin, Snail, Twist) and transcription factors such as NF-κB and HIF-1α, induced by co-infection, may indicate malignant transformation. The concurrent detection of microbial nucleic acids and host biomarkers in saliva or gingival crevicular fluid could offer a promising approach for the early identification of high-risk individuals.

## 7. Conclusions and Future Directions

This review suggests that *P. gingivalis* and HCMV may cooperate in modulating a host’s immune response, inducing ROS and tissue damage, and disrupting the blood coagulation system, all of which contribute to chronic inflammation and PD progression. Additionally, the synergistic interaction between *P. gingivalis* and HCMV may interfere with key cellular processes such as cell proliferation, apoptosis, EMT, and cell immortalization, contributing to carcinogenesis.

Despite these findings, a lot of questions remain unresolved. A critical gap concerns the temporal sequence of infection. *P. gingivalis* may act as an initiator, disrupting epithelial integrity and allowing for a subsequent HCMV infection, which could prolong inflammation and facilitate immune evasion. Alternatively, HCMV may precede bacterial colonization, particularly in immunocompromised hosts, thereby establishing conditions that favor pathogenic overgrowth. This aligns with a possible “second-hit” model, though longitudinal studies are urgently needed to elucidate the order of events and their role in disease progression.

From a clinical standpoint, identifying co-infection by *P. gingivalis* and HCMV in PD may help clinicians stratify patients at higher risk for disease progression and potential transition to oral cancer (OC). In this context, combination therapies integrating antimicrobial and antiviral agents may offer improved treatment outcomes [204,205]. Additionally, preventive strategies are gaining momentum. The ongoing research includes vaccine candidates targeting *P. gingivalis* fimbriae and outer membrane proteins, alongside HCMV subunit and mRNA-based vaccines, some of which have progressed to phase II and III clinical trials [206,207].

On the other hand, future research should focus on several key areas: (1) Mechanistic studies on the participation of oncogenic signaling pathways including NF-κB, PI3K/AKT, and MAPK, which *P. gingivalis* and HCMV could use to induce tumorigenesis. (2) Longitudinal clinical studies evaluating the correlation between persistent periodontal co-infections and the incidence of oral premalignant or malignant lesions, particularly in high-risk populations. (3) An exploration of the immune-evasive strategies employed by both pathogens to persist in mucosal environments, which may reveal targets for next-generation immunotherapies or mucosal vaccines. (4) An evaluation of vaccine candidates targeting HCMV latency and reactivation, especially in populations with high CMV seroprevalence, where re-infection and viral persistence may amplify chronic inflammation and oncogenic risk.

Consequently, including clinical surveillance with molecular and immunological profiling could open the path for precision strategies in the prevention and management of pathogen-associated oral cancers.

## Figures and Tables

**Table 1:** Role of *P. gingivalis* and HCMV co-infection in periodontal disease.

Reference	Disease	Results
Nakamura et al. (2020) [44]	Aggressive periodontitis	The co-presence of *P. gingivalis* and HCMV was associated with periodontal pockets ≥ 4 mm deep with BOP (*p* = 0.03).
Michalowicz et al. (2020) [43]	Juvenile periodontitis	The presence of *P. gingivalis* was significantly associated with HCMV infection (OR = 3.6, 95% CI: 1.5–8.8; *p* = 0.0054). *P. gingivalis*/HCMV co-infection was associated with the extent of attachment loss (*p* < 0.05).
Joshi et al. (2016) [58]	Chronic periodontitis	An increased level of *P. gingivalis* was evidenced by culture in HCMV-positive patients (*p* = 0.023).
Botero et al. (2007) [57]	Aggressive periodontitis	Co-infection with HCMV and periodontopathic bacteria, including *P. gingivalis*, increases the risk of periodontitis development (OR = 7.6, 95% CI: 6.31–9.28; *p* < 0.01).
Elamin et al. (2017) [45]	Aggressive periodontitis	*P. gingivalis* and HCMV co-infection increases the risk of developing aggressive periodontitis (OR = 29.3, 95% CI: 3.1–278.8; *p* = 0.003).
Imbronito et al. (2008) [31]	Aggressive periodontitis	*P. gingivalis* and HCMV co-detection was more frequent in aggressive periodontitis sites than in healthy controls without statistically significant difference among groups
Saygun et al. (2004) [59]	Aggressive periodontitis	HCMV and *P. gingivalis* co-infection occurred in 77.8% of patients with aggressive periodontitis and was significantly more frequent than in healthy individuals (*p* < 0.004).
Herrero-Sánchez et al. (2025) [60]	Stage I-II Periodontitis	No significant association was found between the presence of *P. gingivalis* and HCMV in aggressive periodontitis patients (*p* = 0.732).
Slots et al. (2003) [61]	Aggressive periodontitis	HCMV and HSV were significant predictors of subgingival *P. gingivalis* presence. Their co-detection was associated with increased periodontitis disease activity (*p* < 0.001).
Sharma et al. (2015) [62]	Aggressive periodontitis	Co-infection with *P. gingivalis* and HCMV/EBV-I was observed in 40% and 50% of patients, respectively, but without significant association. However, a strong association (77.78%, *p* = 0.0002) was observed between Aa and HCMV.
Kousar et al. (2017) [63]	Chronic periodontitis	*P. gingivalis* was prevalent in 50–60% of moderate and severe chronic periodontitis patients, but HCMV was not detected in any clinical group.
Passariello et al. (2017) [64]	Aggressive periodontitis	HCMV was inversely associated with *P. gingivalis*, *Tannerella forsythia*, and *Fusobacterium periodonticum* (*p* < 0.05). This suggests that HCMV may influence the subgingival biofilm composition in a virus-specific manner, possibly reducing *P. gingivalis* prevalence.
Kamma et al. (2001) [65]	Early-onset periodontitis	HCMV was detected in 59.4% of active sites and 12.5% of stable sites (*p* < 0.001). *P. gingivalis* was present in 71.9% of active sites and 37.5% of stable sites (*p* = 0.01). Co-infection with HCMV and *P. gingivalis* (along with *D. pneumosintes*) occurred in 60% of herpesvirus-positive sites and only 10.3% of virus-negative sites (*p* = 0.03).
Rams et al. (2024) [66]	Aggressive periodontitis (Ag/MI)	*P. gingivalis* was found in 63.6% of Ag/MI periodontitis patients. Herpesvirus co-infection (CMV + EBV-1) was present in generalized Ag/MI periodontitis cases and absent in healthy controls. Herpesvirus presence showed a 3.5-fold increased odds of Ag/MI periodontitis.
Contreras et al. (1999) [67]	Adult periodontitis	HCMV was associated with co-infections involving *P. gingivalis*, especially with *P. nigrescens* and *T. denticola* (ORs = 2.59–3.23). Mixed viral infections increased odds of severe periodontitis (OR = 4.36) and were associated with *P. gingivalis* (OR = 2.27) and various bacterial combinations (ORs up to 2.91).

Legend: HCMV, Human cytomegalovirus; BOP, Bleeding on probing; OR, Odd ratio.

## Data Availability

Not applicable.

## Supplementary Materials

The following supporting information can be downloaded at https://www.mdpi.com/article/10.3390/cancers17091525/s1: Supplementary S1: Literature search strategy.

## Abbreviations

The following abbreviations are used in this manuscript:

4NQO	4-nitroquinoline-1 oxide
ABL	Alveolar bone loss
aHR	Adjusted hazard ratio
aOR	Adjusted odd ratio
BOP	Bleeding on probing
CAL	Clinical attachment loss
CASP	Caspase
CCL5	CC motif ligand 5
CI	Coefficient interval
CK	Cytokeratin
COX-2	Cyclooxygenase-2
CXCL2	CXC motif chemokine ligand 2
CXCL11	CXC motif chemokine ligand 11
DCs	Dendritic cells
DMFT	Decayed, missing, and filled teeth
EBV	Epstein-Barr virus
EMT	Epithelial–mesenchymal transition
EpCAM	Epithelial cell adhesion molecule
FISH	Fluorescence in situ hybridization
GBD	Global burden of disease
H2S	Hydrogen sulfide
HDI	Human development index
HCMV	Human cytomegalovirus
HHV-5	Human Herpesvirus 5
HNSCC	Head and neck squamous cell carcinoma
HPV	Human papillomavirus
HSV-1	Herpes simplex virus-1
hTERT	Human telomerase reverse transcriptase
HUVECs	Human umbilical vein endothelial cells
IFI16	Gamma-IFN-inducible protein 16
IHC	Immunohistochemistry
IHGE	Immortalized human gingival epithelial cells
IL	Interleukin
INF-γ	Interferon-gamma
IRAK IL	1R-associated kinase
IRF	Interferon regulatory factor
ISH	in situ hybridization
JAK1	Janus kinase
LPS	Lipopolysaccharide
MAPKs	Mitogen-activated protein kinases
MDSCs	Myeloid-derived suppressor cells
MHC	Major histocompatibility complex
miR	microRNA
MMP	Matrix metalloproteinase
MOAP1	Modulator of apoptosis-1
mtDNA	mitochondrial DNA
mtROS	mitochondrial ROS
MyD88	Myeloid differentiation factor 88
Nmi	Human N-myc interactor
NLRX1	Nucleotide-binding oligomerization domain, leucine rich repeat containing X1
OC	Oral cancer
OMVs	Outer membrane vesicles
OR	Odd ratio
OSCC	Squamous cell carcinoma
PAI-1	Plasminogen activator inhibitor type I
PD	Periodontal disease
PI3K	Phosphoinositide 3-kinase
PKC	Protein kinase C
PPD	Probing pocket depth
proMMP	pro matrix metalloproteinase
RNS	Reactive nitrogen species
ROS	Reactive oxygen species
SCFAs	Short-chain fatty acids
SMD	Standardized mean difference
TAMs	Tumor-associated macrophages
TANs	Tumor-associated neutrophils
TCGA	The Cancer Genome Atlas
TLR	Toll-like receptor
TNF-α	Tumor necrosis factor-alpha
TSP-1	Thrombospondin-1
