# Supplementary figures and images for: Porphyromonas gingivalis and Human Cytomegalovirus Co-Infection: A Potential Link Between Periodontal Disease and Oral Cancer Development
Source: Cancers (Basel). 2025 Apr 30;17(9):1525. doi: 10.3390/cancers17091525 (PMC12071019; doi:10.3390/cancers17091525)

## Supplementary S1. Literature search strategy

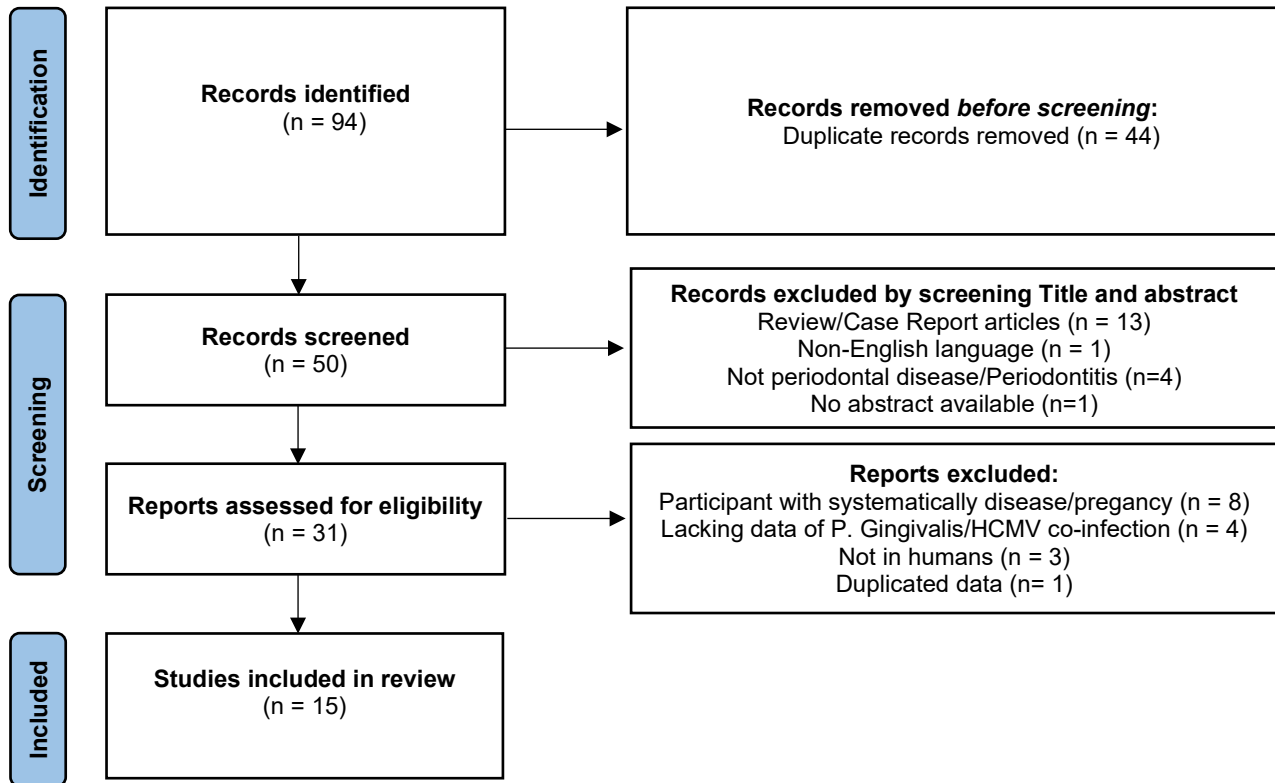

Supplement: Supplementary file 1 [file cancers-17-01525-s001.zip › cancers-3603342-supplementary.pdf]
